# Effects of Electron Beam Radiation on the Phenolic Composition and Bioactive Properties of Olive Pomace Extracts

**DOI:** 10.3390/antiox13050558

**Published:** 2024-05-01

**Authors:** Joana Madureira, Inês Gonçalves, Jéssica Cardoso, Maria Inês Dias, Pedro M. P. Santos, Fernanda M. A. Margaça, Celestino Santos-Buelga, Lillian Barros, Sandra Cabo Verde

**Affiliations:** 1 Centro de Ciências e Tecnologias Nucleares (C2TN), Instituto Superior Técnico, Universidade de Lisboa, E.N. 10 ao km 139.7, 2695-066 Bobadela LRS, Portugal; joanamadureira@ctn.tecnico.ulisboa.pt (J.M.); inesisabel.goncalves@gmail.com (I.G.); jesscrds@hotmail.com (J.C.); psantos@ctn.tecnico.ulisboa.pt (P.M.P.S.); fmargaca@ctn.tecnico.ulisboa.pt (F.M.A.M.); 2 Centro de Investigação de Montanha (CIMO), Instituto Politécnico de Bragança, Campus de Santa Apolónia, 5300-253 Bragança, Portugal; maria.ines@ipb.pt (M.I.D.); lillian@ipb.pt (L.B.); 3 Laboratório Associado para a Sustentabilidade e Tecnologia em Regiões de Montanha (SusTEC), Instituto Politécnico de Bragança, Campus de Santa Apolónia, 5300-253 Bragança, Portugal; 4 Grupo de Investigación en Polifenoles (GIP-USAL), Facultad de Farmacia, Universidad de Salamanca, Campus Miguel de Unamuno s/n, 37007 Salamanca, Spain; csb@usal.es; 5 Centre for Ecology, Evolution and Environmental Changes (cE3c), Faculdade de Ciências, Universidade de Lisboa, Campo Grande, 1749-016 Lisboa, Portugal; 6 Escola Superior de Tecnologia da Saúde de Lisboa (ESTeSL), Instituto Politécnico de Lisboa, 1990-096 Lisboa, Portugal; 7 Departamento de Engenharia e Ciências Nucleares, Instituto Superior Técnico, Universidade de Lisboa, 2695-066 Bobadela LRS, Portugal; 8 Unidad de Excelencia Producción, Agrícola y Medioambiente (AGRIENVIRONMENT), Parque Científico, Universidad de Salamanca, 37185 Salamanca, Spain

**Keywords:** olive pomace, bioactive compounds, ionizing radiation, valorization, bioactivities

## Abstract

Olive pomace is an agro-industrial waste product generated from the olive oil industry and constituted by bioactive compounds with potential applications in several industrial sectors. The purpose of this work was to evaluate the effects of electron beam (e-beam) radiation on olive pomace, specifically on phenolic compounds (by HPLC–DAD–ESI/MS) and the bioactive properties (antioxidant, antiproliferative, and antimicrobial activities) of crude olive pomace (COP) and extracted olive pomace (EOP) extracts. The amount of total flavonoid content and the reducing power of COP extracts were higher than those obtained for EOP extracts. The results suggested that e-beam radiation at 6 kGy increased both total phenolic and total flavonoid contents as well as the reducing power of COP extracts, due to the higher extractability (>2.5-fold) of phenolic compounds from these samples, while decreasing the scavenging activity of extracts. The extracts of both olive pomaces showed antibacterial potential, and COP extracts at 400 µg/mL also presented antiproliferative activity against A549, Caco-2, 293T, and RAW264.7 cell lines, with both properties preserved with the e-beam treatment. All in all, e-beam radiation at 6 kGy appears to be a promising technology to valorize the pollutant wastes of the olive oil industry through enhancing phenolic extractability and bioactive properties, and, furthermore, to contribute to the environmental and economical sustainability of the olive oil industry.

## 1. Introduction

Olive oil is one of the most consumed products, and it is specially linked to countries in the Mediterranean region countries. During the olive oil extraction process, high quantities of wastes are generated, which is very polluting for the environment if discharged without treatment. These wastes contain high amounts of organic substances, such as sugars, fibers, polyalcohols, volatile fatty acids, pectins, and fats, as well as a variety of phenolic compounds like hydroxytyrosol and tyrosol, secoiridoid derivatives, phenolic acids, and flavonoids [1,2], thus constituting a valuable resource of natural bioactive compounds. In fact, during the process of olive oil production high amounts of these compounds are retained in the olive pomace waste, and only 1% is detected in the olive oil [3].

Several conventional and emerging extraction technologies have been used to recover the phenolic compounds from olive pomace, including maceration [1,4,5,6], centrifugation [7], hydrothermal treatment [8], membrane technologies [9], superheated liquid extraction [10], ultrasound-assisted extraction [11], microwave-assisted extraction [12], pressurized liquid extraction [13], supercritical fluid extraction [14], and multi-frequency-multimode-modulated-ultrasonic processing [15]. The authors previously identified hydroxytyrosol as the main phenolic compound present in olive pomaces along with high amounts of hydroxytyrosol-1-β-glucoside, tyrosol, luteolin-7-O-rutinoside, and verbascoside [1]. Nunes et al. [15] also reported hydroxytyrosol as the most abundant (54%) compound in olive pomace, followed by comselogoside (25%), elenolic acid derivative (6%), and tyrosol (3%). On the other hand, Suárez et al. [7] described oleuropein aglycones and mono- and di-aldehydes as the main compounds in solid residues of the olive industry, together with elenolic acid, apigenin-7-glucoside, hydroxytyrosol, and verbascoside.

Currently, there is a growing interest of customers in demanding and consuming healthier food, as well as in using new natural ingredients to replace synthetic ones. In this respect, phenolic compounds extracted from olive pomace are promising candidates, as they have been reported to possess antioxidant, antimicrobial, and antiproliferative activities, among others [16,17,18,19,20].

Ionizing radiation is a safe and eco-friendly technology that avoids the addition of chemicals. This technology is currently used for various applications, including sterilization of medical devices as a substitute for ethylene oxide treatment [21]; preparation and functionalization of hybrid materials that are used for biomedical applications and food packaging [22]; heritage preservation, such as parchment documents [23]; wastewater treatment [24,25]; and food irradiation for preservation [26]. There are numerous ionizing radiation facilities around the world currently operating. Furthermore, this technology has been effective in enhancing the extraction and/or the bioactive properties of some of the chemical compounds present in food, wastes, and plants. Previous works reported an increase in the total phenolic content and/or antioxidant activity of fruits such as strawberries [26], raspberries [27], cherry tomatoes [28], and chestnuts [29]. Similar results have been found in industrial cork wastewaters [30] and aromatic plants [31]. Furthermore, the authors found that low doses of gamma radiation (5 kGy) significantly enhanced the extractability of phenolic compounds and the antioxidant activity of the extracted olive pomace [1].

To our knowledge, there are no studies regarding the use of electron beam (e-beam) radiation with the aim of improving the bioactive properties of agro-industrial wastes. In fact, there are extraction methodologies that have been described as improving the bioactivity of olive pomace [11,12,32]. Nevertheless, a limitation of the industrial use of natural products is their low yields by conventional and non-conventional methods, and ionizing radiation can be used as a pretreatment to improve extraction yields. In this way, this work was intended to evaluate the effects of e-beam treatment on the extraction of bioactive compounds from olive pomace and on their bioactive properties, namely antioxidant, antimicrobial, and antiproliferative activities. Furthermore, the phenolic profile of the obtained extracts was characterized to try to ascertain the possible contribution of the individual compounds to the observed effects. It is expected that these findings can contribute to valorizing the wastes of the olive oil industry and increase the sustainability of the sector, both economically and environmentally.

## 2. Materials and Methods

### 2.1. Olive Pomace Samples

Olive pomace was sampled from UCASUL—União de Cooperativas Agrícolas do Sul—in the Alentejo region of Portugal. Two different types of samples were collected: non-defatted or crude olive pomace (COP) and defatted or extracted olive pomace (EOP), as described by Madureira et al. [1].

### 2.2. Irradiation Experiments

Irradiation experiments were carried out in a linear electron accelerator (LINAC, adapted from GE Saturne 41) with an energy of 10 MeV, located at the Instalação de Radiações IonizanteS (IRIS) of the Centro de Ciências e Tecnologias Nucleares (C2TN) of Instituto Superior Técnico, Universidade de Lisboa (Portugal). COP and EOP samples (30 g in sealed bags) were irradiated at room temperature at 6.0 ± 0.1 and 10.8 ± 0.4 kGy using a dose rate of 1.5 kGy/min. The absorbed doses were estimated using calibrated radiochromic dosimeters FWT-60 (Far West Technology, Inc., Goleta, CA, USA) [33] (dose uniformity DUR = 1.2). The irradiations were performed in triplicate. In order to simplify the discussion of the results, the doses will be referred to 6 and 11 kGy. To verify the effects the of e-beam on olive pomace, non-irradiated (0 kGy) samples were used as controls.

### 2.3. Phenolic Compounds Extraction

After irradiation, a solid–liquid extraction method was used to prepare the olive pomace extracts as previously described [1] using an ethanol:water mixture (80:20, *v*/*v*; 30 mL) as solvent and a total extraction time of 2 h. After this extraction, the ethanol was evaporated (rotary evaporator Büchi R-210, Flawil, Switzerland). Dry extracts were obtained by freeze-drying the aqueous phase.

### 2.4. Evaluation of Bioactive Properties

#### 2.4.1. Total Phenolic Content (TPC)

The total phenolic contents of both EOP and COP extract solutions (1.25 mg/mL) were determined using the Folin-Ciocalteu method [34] with some modifications [26]. The absorbance of the reaction mixture was measured at 765 nm using a Shimadzu UV 1800 (Kyoto, Japan) spectrophotometer. Analyses were carried out in triplicate, and the results were expressed as mg of gallic acid equivalents (GAE) per g of olive pomace extract.

#### 2.4.2. Total Flavonoid Content (TFC)

Total flavonoid content was determined using the Aluminum Chloride Colorimetric method, as previously described by Barkaoui et al. [35], using EOP and COP extract solutions at 1.25 mg/mL. The absorbance of the resultant solution was measured at 510 nm in a spectrophotometer (Shimadzu UV 1800). A standard curve was prepared using catechin and the results were expressed as mg of catechin equivalents (CAE) per g of olive pomace extract. Analyses were performed in triplicate.

#### 2.4.3. Antioxidant Activity

Antioxidant activity was evaluated by two different assays: in vitro 2,2-Diphenyl-1-picrylhydrazyl (DPPH) radical scavenging activity, as previously described by Barkaoui et al. [26], and ferric reducing antioxidant power (FRAP) as described by Benzie and Strain [36], with some modifications [26].

For the FRAP assay, COP and EOP extracts were dissolved in distilled water at a concentration of 0.625 mg/mL. For the DPPH assay, solutions of COP and EOP extracts were prepared in distilled water at a concentration of 10 mg/mL and successively diluted (from 5000 to 39 µg/mL). Both assays were performed in triplicate.

#### 2.4.4. Antimicrobial Activity

##### Antibacterial Activity

Measurement of antibacterial activity by a microdilution method was performed as described by Madureira et al. [1] using three Gram-negative bacteria, *Escherichia coli* (ATCC 8739), *Pseudomonas fluorescens* (ATCC 13525) and *Salmonella enterica* serotype Typhimurium (ATCC 14028), and four Gram-positive bacteria: *Staphylococcus aureus* (ATCC 6538), *Bacillus cereus* (SSI C1/1), *Enterococcus faecalis* (ATCC 29212), and *Listeria monocytogenes* (ATCC 19111). Extract concentrations (10–60 mg/mL for Gram-positive bacteria and 20–100 mg/mL for Gram-negative bacteria) were set up directly in the microplate.

##### Antifungal Activity

Three fungi were used in the antifungal activity assessment: *Candida albicans* (ATCC 10231), *Aspergillus fumigatus* (environment isolate), and *Aspergillus* section *Nigri* (environment isolate). The assay was carried out as described by Madureira et al. [1]. Different concentrations (20–100 mg/mL) were prepared directly in the microplate well.

#### 2.4.5. Cytotoxicity Assay—WST-1 Proliferation Test

Cell viability was assessed using the WST-1 cell proliferation assay, according to the protocol described by Madureira et al. [30] and Barkaoui et al. [26]. For this assay, human lung carcinoma epithelial cells (A549, ATCC^®^ CCL- 185™), human colon adenocarcinoma epithelial cells (Caco-2, ATCC^®^ HTB-37™), human embryonic kidney epithelial cells (293T, ATCC^®^ CRL-3616™), and mouse monocyte macrophage cells (RAW264.7, ATCC^®^ TIB-71™) were used. As the best results for antioxidant activity were obtained using COP extracts, the antiproliferative potential was evaluated for these extracts (from irradiated and non-irradiated samples).

### 2.5. Analysis of Phenolic Compounds

The dry EOP and COP extracts (∼10 mg) were dissolved in an ethanol:water mixture (20:80 *v*/*v*, 2 mL) and filtered with 0.22 µm disposable LC filter disks. The extracts were examined by HPLC–DAD–ESI/MSn (Dionex Ultimate 3000 UPLC, Thermo Scientific, San Jose, CA, USA) as described by Bessada et al. [37]. The phenolic compounds were identified by considering the following: (i) chromatograms and UV–vis and mass spectra; (ii) comparison with standard compounds, when available; and (iii) data reported in the literature [1,38,39,40,41]. The results were expressed in mg per g of olive pomace extract and the analyses were carried out in triplicate.

### 2.6. Statistical Analysis

Data results were presented as mean ± standard error. In the data analyses, standard errors for mean values were estimated using a significance level of *p* < 0.05 and the number of replicates for each assay. The results were evaluated using the one-way analysis of variance (ANOVA) test followed by Tukey’s HSD test with α = 0.05.

## 3. Results and Discussion

As mentioned before, two types of olive pomace—crude olive pomace (COP) and extracted olive pomace (EOP)—were analyzed to identify the best conditions for the extraction of their phenolic compounds and for improving the bioactive properties of both extracts.

### 3.1. Bioactive Properties of Olive Pomace Extracts

#### 3.1.1. Total Phenolic Content (TPC) and Total Flavonoid Content (TFC)

TPC and TFC were the first measures to be determined because they provide rapid measures to assess the amounts of potential bioactive compounds in the pomace extracts (Table 1).

For non-irradiated samples, no significant differences were observed in TPC between COP (71 ± 1 mg GAE/g extract) and EOP (67.8 ± 0.8 mg GAE/g extract) extracts. After e-beam radiation, a significant increase was detected in the TPC of COP extracts from olive pomace irradiated at 6 kGy (95 ± 3 mg GAE/g extract) and at 11 kGy (86 ± 2 mg GAE/g extract). On the other hand, for EOP extracts, no significant variations were observed in TPC at the different doses of irradiation assayed (60 ± 2 mg GAE/g extract at 6 kGy and 70 ± 3 mg GAE/g extract at 11 kGy).

Concerning TFC for non-irradiated samples, the values obtained for COP extracts (131 ± 1 mg CAE/g extract) were significantly higher than those for EOP extracts (115 ± 1 mg CAE/g extract) (Table 1). Furthermore, as in TPC, the same trend was observed in TFC for both extracts of EOP and COP after exposure to e-beam radiation. An increase of TFC was noticed in COP extracts from samples irradiated at 6 kGy (143 ± 1 mg CAE/g extract), whereas values at 11 kGy (132 ± 2 mg CAE/g extract) where similar to the values of non-irradiated samples. In either case, the higher values of TFC and TPC were obtained for COP samples irradiated at 6 kGy.

Gómez-Cruz et al. [42] characterized the non-irradiated exhausted olive pomace from a local olive pomace factory in Spain, and the results reported a similar value of TPC (11.5 ± 0.1 mg GAE/g olive pomace) to that obtained in this study (67.8 ± 0.8 mg GAE/g extract, corresponding to 9.9 ± 0.1 mg GAE/g olive pomace). Furthermore, Shalaby et al. [43] detected significantly lower contents of TPC (3.88 mg GAE/g extract) and TFC (2.99 mg QE/g extract) in non-irradiated olive leaves than those in this study, and they observed an increase in these parameters when the leaves were subjected to gamma radiation, especially at 10 kGy. As far as the authors know, there are no studies reporting the effects of e-beam radiation on TPC and TFC extractability from olive pomace. The increase in levels of phenolic compounds in extracts from irradiated samples could be attributed to changes in the plant’s cellular structure. Specifically, this could be due to the release of fractions associated with polysaccharides and other matrix components, or to the degradation of larger compounds into smaller ones by e-beam radiation, thus improving the extractability of these compounds [44,45].

#### 3.1.2. Antioxidant Activity

The antioxidant activities of non-irradiated and irradiated olive pomace extracts were evaluated by DPPH scavenging activity and FRAP assays (Table 1).

For FRAP assays, extracts from non-irradiated EOP and COP presented no significant differences in antioxidant activity (1.40 ± 0.02 mmol FES/g extract and 1.46 ± 0.01 mmol FES/g extract for EOP and COP, respectively), which agrees with the TPC values in these samples. Regarding e-beam irradiation, no significant variation was observed in EOP extracts, whereas for COP extracts the antioxidant activity was significantly increased at 6 kGy (1.78 ± 0.04 mmol FES/g extract), again in agreement with the observation made for TPC in these samples. This outcome can be considered logical considering that the Folin-Ciocalteu method is a measurement of total reducing substances (as is the FRAP method).

DPPH results are expressed as IC_50_ values that represent the extract concentrations providing a 50% level of DPPH radical scavenging activity. Non-irradiated COP extracts showed higher scavenging activity (IC_50_ value of 480 ± 9 µg/mL) than non-irradiated EOP extracts (IC_50_ value of 561 ± 9 µg/mL). Interestingly, e-beam radiation promoted an increase in the scavenging activity of EOP extracts (lower IC_50_ values: 440 ± 9 µg/mL at 6 kGy and 462 ± 5 µg/mL at 11 kGy) to values similar to those of non-irradiated COP extracts. On the other hand, the scavenging activity in COP extracts was significantly decreased at 6 kGy (IC_50_ value of 545 ± 13 µg/mL). Similar results were obtained by Madureira et al. [1] with samples treated by gamma radiation and analyzed through the thiobarbituric acid reactive substances (TBARS) assay. In that work, the authors attributed this trend to the fat content present in these samples that could form a barrier and inhibit contact between the antioxidant compounds and the radicals generated during irradiation.

There are no studies in the literature reporting the effect of e-beam radiation on the antioxidant activity of olive pomace. A study using gamma-irradiated olive leaf extracts to improve the quality and shelf-life of minced beef reported that all studied irradiation doses (5, 10 and 15 kGy) promoted increases in antioxidant activity by DPPH and FRAP assays, with the highest value observed for 10 kGy-irradiated samples [43]. This increase in antioxidant activity might be related to an enhancement of phenylalanine ammonia-lyase (PAL) activity that is induced by the irradiation process, as suggested by Hussain et al. [46], which promotes the accumulation of phenolic compounds, thus increasing the antioxidant potential of olive pomace samples.

#### 3.1.3. Antimicrobial Activity

The antimicrobial activity of COP and EOP extracts was evaluated against three Gram-negative bacteria (*E. coli*, *P. fluorescens* and *S.* Typhimurium), four Gram-positive bacteria (*S. aureus*, *B. cereus*, *E. faecalis* and *L. monocytogenes*), and three fungi (*C. albicans*, *A. fumigatus* and *A.* section *Nigri*) (Table 2).

The obtained results suggested that both olive pomace extracts (COP and EOP) inhibited the growth of the studied Gram-negative bacterial strains (MIC 60 mg/mL). Regarding Gram-positive bacteria, COP samples showed the strongest antimicrobial activity against *S. aureus* (MIC 20 mg/mL), whereas for *L. monocytogenes* (MIC 20 mg/mL), *B. cereus* (MIC 20 mg/mL), and *E. faecalis* (MIC > 60 mg/mL), there was no difference between COP and EOP extracts. The overall results pointed out that olive pomace extracts were more effective against Gram-positive bacteria than against Gram-negative bacteria, which was also reported by Madureira et al. [1] for olive pomace and Brenes et al. [47] for olive oil. In agreement with these results, Madureira et al. [1] also observed the same MIC values (MIC 20 mg/mL) for extracts from both gamma-irradiated COP and gamma-irradiated EOP against the studied Gram-positive bacteria (*B. cereus*, *S. aureus* and *L. monocytogenes*), with exception of EOP against *S. aureus*. Concerning the Gram-negative bacteria, the results were not in agreement with previously reported results, which might be due to the composition of the samples. MIC is the lowest concentration of an antibacterial agent necessary to inhibit the visible growth of bacteria (to be bacteriostatic), whereas MBC is the minimum concentration of an antibacterial agent required to prevent bacterial viability, and the closer the MIC is to the MBC, the more bactericidal is the compound. In this respect, as for the obtained results (Table 2), it is possible to deduce that COP extracts showed greater bactericidal activity than did EOP extracts, especially against Gram-negative bacteria.

Concerning e-beam radiation, no effect was observed on the antibacterial activity of the studied olive pomace extracts (COP and EOP), which was in accordance with previous results described by Madureira et al. [1] using gamma radiation. Although no studies described the effect of e-beam radiation on olive pomace, there are some works reporting relevant antimicrobial activities in table olives [48,49], olive leaves [50,51], olive oil [47], and olive mill wastewaters [52]. Shalaby et al. [43] observed that gamma irradiation of olive leaf increased the antibacterial activity of the obtained extracts, which can be explained by the increase in their contents of phenolic compounds, with doses of 10 kGy providing the highest inhibition of *Bacillus subtilis*, *S. aureus*, *E. coli*, *Klebsiella pneumonia*, *Pseudomonas aeruginosa*, and *S.* Typhymurium.

For the antifungal activity, the obtained results indicated that none of the extracts from EOP or COP, irradiated or non-irradiated, could inhibit the growth of the three evaluated fungi (MIC and MFC >100 mg/mL) (Table 2), suggesting no antifungal activity of the extracts at the studied concentrations. Contrary to these results, previous works reported the antifungal potential of olive pomace [1] and olive leaves, especially against *C. albicans* [50].

These results suggested that olive pomace extracts could be used as foodborne antimicrobial agents to delay food spoilage. Further studies should, however, be carried out to determine the relationship between the compounds and the antibacterial activity of these samples.

#### 3.1.4. Antiproliferative Activity

**Figure 1:**
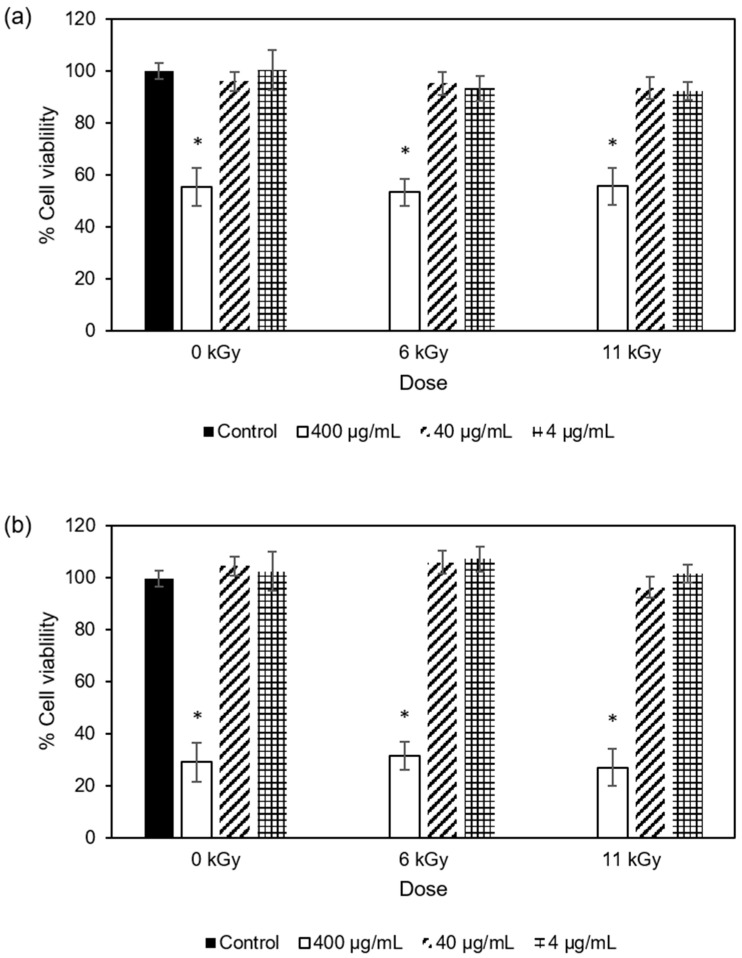
Cellular viability of (**a**) A549 and (**b**) Caco-2 cell lines in the presence of extracts from non-irradiated COP and COP irradiated at 6 kGy or 11 kGy at three different concentrations (400 μg/mL, 40 μg/mL, and 4 μg/mL). The control is used for comparison and does not contain any extract. Each bar graph represents the mean and 95% confidence interval from three separate experiments. For each cell line, bars with * indicate a statistically significant difference from control at *p* < 0.05.

**Figure 2:**
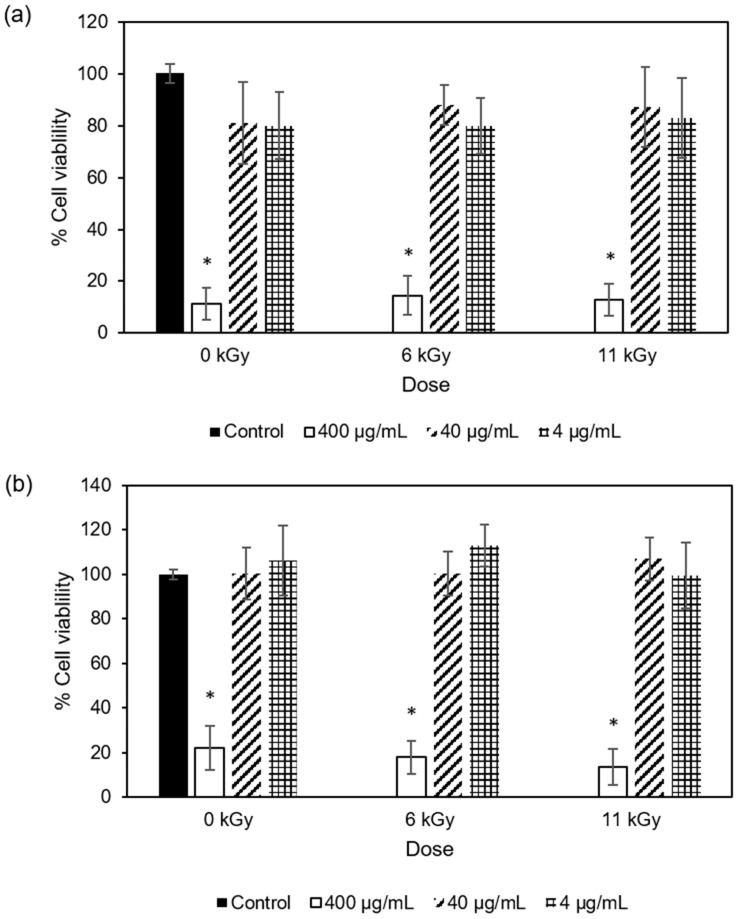
Cellular viability of (**a**) 293-T and (**b**) RAW 264.7 cell lines in the presence of extracts from non-irradiated COP and COP irradiated at 6 kGy or 11 kGy at three different concentrations (400 μg/mL, 40 μg/mL, and 4 μg/mL). The control is used for comparison and does not contain any extract. Each bar graph represents the mean and 95% confidence interval from three separate experiments. For each cell line, bars with * indicate a statistically significant difference from control at *p* < 0.05.

Antiproliferative assessment was only performed for COP extracts as they were those that showed higher phenolic and flavonoid contents and antioxidant activities, as well as showing bactericidal potential for Gram-positive bacteria. Four different cell lines were employed for these assays; two of them were tumoral cells, namely human lung carcinoma epithelial cells (A549) and human colon adenocarcinoma epithelial cells (Caco-2) (Figure 1), and the other two non-tumoral cells, namely human embryonic kidney epithelial cells (293T) and mouse monocyte macrophage cells (RAW 264.7) (Figure 2).

As observed in Figure 1 and Figure 2, the highest extract concentration (400 μg/mL) significantly decreased the viability of both tumor and non-tumor cells by 44–89%, suggesting that the extracts could be toxic to the cells at these concentrations (at least ≥400 μg/mL) and indicating that the extract concentrations 4–40 μg/mL did not affect the viability of the analyzed cells. Despite the differences in phenolic contents, no significant differences were observed between non-irradiated and e-beam-irradiated samples (6 and 11 kGy).

As far as the authors know, there are no reported studies into the antiproliferative activity of non-irradiated and irradiated olive pomace extracts against the assayed cell lines. Nonetheless, other authors studied the cytotoxic potential of different olive waste products in other tumor cells [53,54,55]. Taamalli et al. [55] assessed the antiproliferative activity of six different samples of Tunisian olive leaves against a human breast carcinoma cell line (JIMT-1), observing that their cytotoxicity seemed to be more related to the type of phenolic compounds present than to the total extraction yield. Reboredo-Rodríguez et al. [54] reported a significant decrease in the viability of breast cancer cells (MCF-7) treated with extracts of extra virgin olive oils from the ‘Brava’ cultivar. Anter et al. [53] demonstrated that “alperujo” olive pomaces and their three most abundant phenolic compounds (hydroxytyrosol, tyrosol, and verbascoside) induced an antimutagenic effect and the death of HL60 human promyelocytic leukemia cells. More recently, Madureira et al. [56] also observed antiproliferative effects of olive pomace extracts against a breast adenocarcinoma (MCF-7) cell line.

### 3.2. Phenolic Profile Characterization

**Figure 3:**
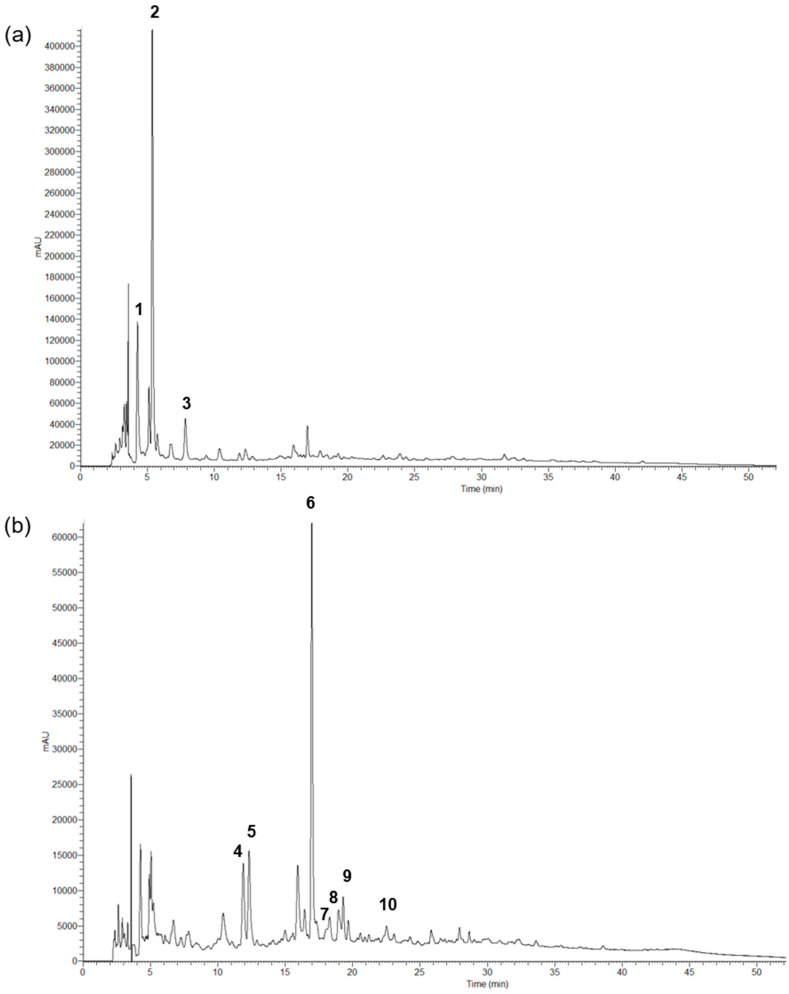
Chromatographic profile of the hydroethanolic extracts prepared from non-irradiated EOP samples, recorded at (**a**) 280 nm and (**b**) 370 nm.

Similar qualitative phenolic profiles were observed for EOP and COP samples, either non-irradiated or irradiated. As an example, the phenolic profiles recorded at 280 and 370 nm of an extract from non-irradiated COP samples is shown in Figure 3.

The phenolic compounds present in the extracts were characterized and tentatively identified by their UV–vis and MS spectra and comparison with the literature. In both EOP and COP samples, nine phenylethanoid derivatives (peaks 1, 2, 3, 4, 5, 6, 8, 9, and 10) and one flavonoid (peak 7) were detected (Table 3).

Hydroxytyrosol-1-β-glucoside (peak 1), hydroxytyrosol (peak 2), tyrosol (peak 3), β-hydroxyverbascoside isomer 1 (peak 4), β-hydroxyverbascoside isomer 2 (peak 5) verbascoside (peak 6) and luteolin-7-*O*-rutinoside (peak 7) were identified by comparison with standards. All of these compounds were previously described in olive pomaces [1], olive wood and leaves [38], and olive mill wastewaters [39,41]. Peak 8 exhibited a pseudomolecular ion [M−H]^−^ at *m*/*z* 701, 162 mu higher than oleuropein (Table 3), which could indicate the presence of a hexose moiety. Furthermore, its fragmentation pattern was consistent with that of oleuropein, with fragment ions at *m*/*z* 377 (loss of a hexose moiety), 307 (loss of C_4_H_6_O group), and 275 (loss of CH_3_OH group). Based on this, peak 8 was identified as oleuropein-*O*-hexoside, whose presence in olive leaf extracts was recently described [40]. Peak 9 presented the same fragmentation as verbascoside (Table 3) and, by comparison of their retention times, it could be interpreted as isoverbascoside, a compound also reported by Cardinali et al. [39] and Ammar et al. [38] in Olea europaea by-products. Peak 10 could be assigned to an acetyl derivative of verbascoside owing to its pseudomolecular ion [M−H]^−^ at *m*/*z* 665, which is 42 mu higher than that of verbascoside. To the best of the author’s knowledge, this is the first time that this compound has been described in olive pomace extracts.

In non-irradiated samples, the individual amounts of hydroxytyrosol-1-β-glucoside, hydroxytyrosol, tyrosol, verbascoside, luteolin-7-*O*-rutinoside, oleuropein-*O*-hexoside, and acetylverbascoside derivative were not significantly different (*p* > 0.05) between EOP and COP extracts (Table 4). Nevertheless, COP extracts contained significantly higher levels of β-hydroxyverbascoside isomers 1 and 2, and significantly lower levels of isoverbascoside than did EOP extracts. As expected [1], hydroxytyrosol was the major compound in both samples (11.06 ± 0.43 mg/g extract in EOP samples and 10.1 ± 0.2 mg/g extract in COP samples), followed by hydroxytyrosol-1-β-glucoside, tyrosol, luteolin-7-*O*-rutinoside, oleuropein-*O*-hexoside, and verbascoside. Concerning e-beam radiation effects, different trends were observed for the EOP and COP samples. For COP samples, the extraction of phenolic compounds significantly increased (*p* < 0.05) after e-beam radiation at 6 and 11 kGy. The concentrations of total phenolic compounds in the obtained extracts were 55 ± 7 mg/g and 60 ± 2 mg/g, at 6 and 11 kGy, respectively (Table 4).

Compared to non-irradiated samples, this could represent an increase in extractable phenolic compounds of 2.5 and 2.7 fold, respectively, for 6 and 11 kGy. In fact, in these samples, e-beam radiation significantly improved the extractability of all of the identified compounds, although there were not significantly differences between the applied-dose results. On the other hand, for EOP samples, e-beam radiation seemed to preserve the extractability of the total phenolic compounds. Nevertheless, in these samples, when compared to non-irradiated ones, hydroxytyrosol and isoverbascoside were extracted in lower amounts using an absorbed dose of 11 kGy. The findings from the phenolic compound quantification could explain the obtained results of TPC and FRAP previously discussed. Actually, the higher amounts of phenolic compounds extracted from the COP samples irradiated at 6 kGy (Table 4) could be related to the higher TPC (95 ± 3 mg GAE/g extract) and, consequently, the higher FRAP values (1.78 ± 0.04 mmol FES/g extract) of these samples (Table 1).

To the authors’ knowledge, this is the first study reporting the effects of e-beam radiation on the extractability of olive pomace compounds. In a previous study, Madureira et al. [1] reported that gamma radiation at 5 kGy could be applied to valorize olive oil by-products, although the best results were achieved when defatted samples (EOP) were used. The different results obtained by these studies could be related to the different characteristics of the two irradiation processes. The higher dose rate achieved using e-beam radiation (90 kGy/h, in contrast with 16 kGy/h of gamma radiation), and the reduced penetration of the e-beam could induce changes on the surface of the product, probably breaking the fat barrier of COP samples, which could promote the affinity of the solute with the extraction solvent, thus extracting a greater quantity of phenolic compounds.

Most of the studies in the literature concerning the extraction of phenolic compounds from olive pomace did not quantify the compounds. Even so, comparing the observed results with those reported in the literature using a solid–liquid extraction at atmospheric and high pressures [57] and using microwave irradiation at 400 W [58], it could be possible to demonstrate the potential of using ionizing radiation as a pretreatment to enhance the extraction of individual bioactive compounds from olive pomace (Table 5). In fact, the recovery rates of hydroxytyrosol and tyrosol from crude olive pomace irradiated at 6 kGy are ten times higher than those at high pressure and 260 times higher than those of extraction using microwave irradiation.

## 4. Conclusions

This work evaluated the impact of e-beam radiation on the extractability of phenolic compounds from olive pomace samples, COP and EOP. The amounts of total flavonoid content and the reducing power of COP extracts were higher than those obtained for EOP extracts. The results suggested that e-beam radiation at 6 kGy of crude olive pomaces increased both total phenolic and total flavonoid contents, as well as enhancing the reducing power in the extracts while decreasing its scavenging activity. Both COP and EOP extracts presented antibacterial activity that was preserved by e-beam radiation, suggesting a bactericidal potential (especially for COP extracts) against bacteria commonly associated with food outbreaks. The results from antiproliferative assays indicated that COP extracts at 400 μg/mL decreased the cell viability of A549, Caco-2, 293T, and RAW264.7 cell lines, although no antiproliferative effects were observed at the other extract concentrations, and e-beam radiation of the samples did not induced changes in their effects on cell viability. On the other hand, the extractability of phenolic compounds from COP samples was also increased by 2.5-fold at 6 kGy, which could explain the TPC, TFC, and FRAP values obtained for these samples. Overall, e-beam radiation at 6 kGy seems to be a suitable technology to increase antioxidant activity in olive pomace residues, especially at the crude stage. All in all, the results of the present study could support the possibility of using these extracts as safe bioactive ingredients in value-added food products or supplements. In this way, a residue valorization can be achieved while also decreasing the amounts of residues generated by the olive oil industry and, consequently, their environmental impact.

## Figures and Tables

**Table 1:** Total Phenolic Content, Total Flavonoid Content and antioxidant activity, by DPPH scavenging activity and FRAP assay, of extracts from non-irradiated and irradiated olive pomace. The results are presented as mean ± standard error.

	Total Phenolic Content (mg GAE/g Extract)	Total Flavonoid Content (mg CAE/g Extract)	FRAP (mmol FES/g Extract)	DPPH (IC_50_, µg/mL)
*COP samples*				
0 kGy	71 ± 1 ^b^	131 ± 1 ^b^	1.46 ± 0.01 ^b,c^	480 ± 9 ^b,c^
6 kGy	95 ± 3 ^a^	143 ± 1 ^a^	1.78 ± 0.04 ^a^	545 ± 13 ^a^
11 kGy	86 ± 2 ^a^	132 ± 2 ^b^	1.51 ± 0.02 ^b^	500 ± 10 ^b^
*EOP samples*				
0 kGy	67.8± 0.8 ^b,c^	115 ± 1 ^c,d^	1.40 ± 0.02 ^c,d^	561 ± 9 ^a^
6 kGy	60 ± 2 ^c^	112 ± 1 ^d^	1.41 ± 0.02 ^b,c,d^	440 ± 9 ^c^
11 kGy	70 ± 3 ^b^	118 ± 1 ^c^	1.35 ± 0.02 ^d^	462 ± 5 ^b,c^

GAE—gallic acid equivalents; CAE—catechin equivalents; DPPH—2,2-diphenyl-1-picrylhydrazyl; FRAP—ferric reducing antioxidant power; COP—crude olive pomace; EOP—extracted olive pomace, IC50—extract concentration able to provide 50% of DPPH radical scavenging activity. In each column, means with different letters differ significantly (*p* < 0.05). Non-irradiated samples were used as controls and are reported in 0 kGy rows.

**Table 2:** Antimicrobial activity of the olive pomace extracts (MIC, MBC, and MFC; mg/mL).

	*B. cereus*	*S. aureus*	*L. monocytogenes*	*E. faecalis*	*E. coli*	*S.* Typhymurium	*P. fluorescens*	*A.* section *Nigri*	*A. fumigatus*	*C. albicans*
	MIC (mg/mL)									
COP samples										
0 kGy	20	20	20	>60	60	60	60	>100	>100	>100
6 kGy	20	20	20	>60	60	60	60	>100	>100	>100
11 kGy	20	20	20	>60	60	60	60	>100	>100	>100
EOP samples										
0 kGy	20	>60	20	>60	60	60	60	>100	>100	>100
6 kGy	20	>60	20	>60	60	60	60	>100	>100	>100
11 kGy	20	>60	20	>60	60	60	60	>100	>100	>100
	MBC (mg/mL)							MFC (mg/mL)		
COP samples										
0 kGy	20	40	60	>60	60	100	60	>100	>100	>100
6 kGy	20	40	60	>60	60	100	60	>100	>100	>100
11 kGy	20	40	60	>60	60	100	60	>100	>100	>100
EOP samples										
0 kGy	20	>60	>60	>60	>100	>100	>100	>100	>100	>100
6 kGy	20	>60	>60	>60	>100	>100	>100	>100	>100	>100
11 kGy	20	>60	>60	>60	>100	>100	>100	>100	>100	>100

COP—crude olive pomace; EOP—extracted olive pomace; MIC—Minimum inhibitory concentration; MBC—Minimum bactericidal concentration; MFC—Minimum fungicidal concentration. Non-irradiated samples were used as controls and are reported in 0 kGy rows.

**Table 3:** Chromatographic and mass-spectral-data characteristics and tentative identification of phenolic compounds in extracts obtained from olive pomace samples.

Peak	Rt (min)	λ_max (nm)_	Molecular Ion [M-H] (*m*/*z*)	MS ^2^ (*m*/*z*)	MS ^3^ (*m*/*z*)	Tentative Identification
1	4.26	277	315			Hydroxytyrosol-1-β-glucoside
2	5.38	281	153	123(100)		Hydroxytyrosol
3	7.86	220, 277	137			Tyrosol
4	11.93	278, 324	753	639(100)	621(100), 529(57), 487(62), 459(10)	β-Hydroxyverbascoside isomer 1
5	12.36	282, 321	753	639(100)	621(100), 529(6)	β-Hydroxyverbascoside isomer 2
6	16.99	292, 329	737	623(100)	461(100)	Verbascoside
7	18.96	234, 283, 326	707	593(100)	447(52), 285(100)	Luteolin-7-*O*-rutinoside
8	19.32	234, 284, 329	815	701(100)	377(100), 307(41), 275(29)	Oleuropein-*O*-hexoside
9	19.7	236, 285, 324	737	623(100)	461(100)	Isoverbascoside
10	22.5	239, 281, 323	779	665(100)	623(100), 503(32), 461(49), 443(24)	Acetylverbascoside derivative

**Table 4:** Quantification of phenolic compounds in EOP and COP extracts prepared from non-irradiated and irradiated samples.

Compound	Quantification (mg/g Extract)					
	COP			EOP		
	0 kGy	6 kGy	11 kGy	0 kGy	6 kGy	11 kGy
Hydroxytyrosol-1-β-glucoside ^1^	3.7 ± 0.1 ^b^	9 ± 1 ^a^	9.4 ± 0.4 ^a^	3.6 ± 0.4 ^b^	3.2 ± 0.4 ^b^	2.7 ± 0.3 ^b^
Hydroxytyrosol ^1^	10.1 ± 0.2 ^b,c^	26 ± 2 ^a^	27.1 ± 0.4 ^a^	11.06 ± 0.43 ^b^	9 ± 1 ^b,c^	7.9 ± 0.5 ^c^
Tyrosol ^2^	2.43 ± 0.05 ^b^	6.9 ± 0.3 ^a^	7.7 ± 0.3 ^a^	2.7 ± 0.2 ^b^	2.2 ± 0.1 ^b^	1.95 ± 0.09 ^b^
β-Hydroxyverbascoside isomer 1 ^3^	0.65 ± 0.01 ^b^	1.7 ± 0.1 ^a^	1.8 ± 0.1 ^a^	0.29 ± 0.01 ^c^	0.25 ± 0.02 ^c^	0.2 ± 0.01 ^c^
β-Hydroxyverbascoside isomer 2 ^3^	0.70 ± 0.01 ^b^	1.88 ± 0.08 ^a^	2.08 ± 0.09 ^a^	0.30 ± 0.01 ^c^	0.26 ± 0.02 ^c^	0.25 ± 0.01 ^c^
Verbascoside ^3^	1.73 ± 0.06 ^b^	4 ± 1 ^a^	4.85 ± 0.09 ^a^	1.15 ± 0.03 ^b,c^	0.89 ± 0.08 ^c^	0.81 ± 0.05 ^c^
Luteolin-7-*O*-rutinoside ^4^	1.198 ± 0.003 ^b^	1.76 ± 0.01 ^a^	1.99 ± 0.10 ^a^	1.18 ± 0.01 ^b^	1.12 ± 0.02 ^b^	1.10 ± 0.02 ^b^
Oleuropein-*O*-hexoside ^5^	1.15 ± 0.07 ^c^	2.9 ± 0.2 ^b^	3.7 ± 0.2 ^a^	1.34 ± 0.06 ^c^	1.05 ± 0.08 ^c^	1.05 ± 0.08 ^c^
Isoverbascoside ^3^	0.24 ± 0.01 ^d^	0.546 ± 0.003 ^b^	0.68 ± 0.03 ^a^	0.44 ± 0.01 ^b,c^	0.32 ± 0.03 ^c,d^	0.32 ± 0.02 ^d^
Acetylverbascoside derivative ^3^	0.25 ± 0.02 ^b^	0.6 ± 0.1 ^a^	0.72 ± 0.02 ^a^	0.246 ± 0.001 ^b^	0.20 ± 0.02 ^b^	0.19 ± 0.02 ^b^
Total phenylethanoid derivatives	21 ± 1 ^b^	53 ± 7 ^a^	58 ± 2 ^a^	21 ± 2 ^b^	17 ± 2 ^b^	15 ± 2 ^b^
Total flavonoids	1.198 ± 0.005 ^b^	1.76 ± 0.01 ^a^	1.99 ± 0.17 ^a^	1.18 ± 0.01 ^b^	1.12 ± 0.03 ^b^	1.10 ± 0.04 ^b^
Total phenolic compounds	22 ± 1 ^b^	55 ± 7 ^a^	60 ± 2 ^a^	22 ± 2 ^b^	18 ± 2 ^b^	17 ± 2 ^b^

COP—crude olive pomace; EOP—extracted olive pomace. Values within a row with similar letters do not differ significantly (*p* > 0.05). Calibration curves used for quantification were as follows: ^1^ Hydroxytyrosol (y = 124,154x + 17,393, R^2^ = 0.9999), ^2^ Tyrosol (y = 91,708x + 9398.5, R^2^ = 0.9999), ^3^ Verbascoside (y = 124,233x − 18,873, R^2^ = 1), ^4^ Apigenin-7-O-glucoside (y = 10,683x − 45,794, R^2^ = 0.996), and ^5^ Oleuropein (y = 32,226x + 12,416, R^2^ = 0.9999). Non-irradiated samples were used as controls and are reported in 0 kGy columns.

**Table 5:** Comparison of the results obtained in this study with others reported in the literature (mg/g extract).

	This Study	Suárez et al., 2009 [57]		Sánchez de Medina et al., 2011 [58]
	Crude olive pomace, 6 kGy	Solid–liquid extraction at atmospheric pressure	Solid–liquid extraction at high pressure	Microwave irradiation
Hydroxytyrosol	26	2.79	2.54	0.11
Tyrosol	6.9	0.32	0.09	0.02

## Data Availability

The data presented in this study are available on request from the corresponding author.
